# miR-340-5p Mediates Cardiomyocyte Oxidative Stress in Diabetes-Induced Cardiac Dysfunction by Targeting Mcl-1

**DOI:** 10.1155/2022/3182931

**Published:** 2022-01-27

**Authors:** Yinghong Zhu, Xuewen Yang, Jing Zhou, Long Chen, Pengfei Zuo, Lin Chen, Lan Jiang, Ting Li, Dejiang Wang, Yingyi Xu, Qiushi Li, Yi Yan

**Affiliations:** ^1^Department of Cardiology, The Third Affiliated Hospital of Guangzhou Medical University, Guangzhou 510150, China; ^2^Department of Endocrinology, Sun Yat-sen Memorial Hospital of Sun Yat-sen University, Guangzhou 510120, China; ^3^Department of Cardiology, Zhongda Hospital Affiliated to Southeast University, Nanjing 210009, China

## Abstract

Diabetic cardiomyopathy (DCM) is initially characterized by early diastolic dysfunction, left ventricular remodeling, hypertrophy, and myocardial fibrosis, and it is eventually characterized by clinical heart failure. MicroRNAs (miRNAs), endogenous small noncoding RNAs, play significant roles in diabetes mellitus (DM). However, it is still largely unknown about the mechanism that links miRNAs and the development of DCM. Here, we aimed to elucidate the mechanism underlying the potential role of microRNA-340-5p in DCM in db/db mouse, which is a commonly used model of type 2 DM and diabetic complications that lead to heart failure. We first demonstrated that miR-340-5p expression was dramatically increased in heart tissues of mice and cardiomyocytes under diabetic conditions. Overexpression of miR-340-5p exacerbated DCM, which was reflected by extensive myocardial fibrosis and more serious dysfunction in db/db mice as represented by increased apoptotic cardiomyocytes, elevated ROS production, and impaired mitochondrial function. Inhibition of miR-340-5p by a tough decoy (TUD) vector was beneficial for preventing ROS production and apoptosis, thus rescuing diabetic cardiomyopathy. We identified myeloid cell leukemia 1 (Mcl-1) as a major target gene for miR-340-5p and showed that the inhibition of Mcl-1 was responsible for increased functional loss of mitochondria, oxidative stress, and cardiomyocyte apoptosis, thereby caused cardiac dysfunction in diabetic mice. In conclusion, our results showed that miR-340-5p plays a crucial role in the development of DCM and can be targeted for therapeutic intervention.

## 1. Introduction

Diabetic cardiomyopathy (DCM) is one of the common complications in people with diabetes mellitus (DM), leading to cardiac dysfunction that develops in the absence of other risk factors like hypertension, coronary artery disease, and valvular heart disease [1, 2]. DCM is characterized by several types of structural and functional properties of the left ventricle (LV), including cardiac hypertrophy and fibrosis as well as systolic and/or diastolic contractile dysfunction. Although the etiology of DCM is multifactorial, recent studies have indicated several pathogenic changes including increased advanced glycation end-products, fibrosis, oxidative stress, impaired Ca2+ balance, impaired mitochondrial function, altered myocardial insulin signaling, and elevated apoptotic and necrotic cell death [3]. Multiple epidemiological shreds of evidence have revealed that DCM contributes to mortality in elderly diabetes patients [1]. Moreover, even in individuals with prediabetes, the risk of cardiovascular disease was increased [4]. Thus, it has become important to prevent and reverse adverse cardiac remodeling and DCM. However, the current major strategies to treat DM patients, such as controlling blood glucose levels, fail to reduce long-term cardiovascular events in DCM patients, which explicate the significance of novel strategies for therapies.

MicroRNAs (miRNAs) are a family of small single-stranded noncoding RNAs of approximately 19-24 nucleotides in length that have multiple functions in cancer, nervous development, metabolic disorders such as obesity, and cardiovascular diseases [5]. Mature miRNAs align and bind to the 3′-UTR sequences of their target mRNAs, and they are integrated into the RNA-induced silencing complex (RISC), leading to target mRNA degradation [6]. A recent study has reported that in the hearts of streptozotocin- (STZ-) induced DM mice, 316 significantly changed miRNAs are identified, and interestingly, glycemic control fails to reverse such hyperglycemia-induced miRNA dysregulation in the diabetic hearts, suggesting that the potential association of miRNAs and the development of DCM might be independent of hyperglycemia [7]. Several miRNAs have been reported to be linked to DCM progression, such as miR-21, miR-320, and miR-34a [8–10]. Although miRNAs are known to regulate several pathophysiological changes, the mechanisms are still largely elusive how specific miRNA dysregulation could lead to pathogenesis in the development of DCM.

miR-340-5p, which localized in 5q35, has been identified to contribute to participate in the initiation and progression of several diseases. Recently, miR-340-5p has been revealed that participates in the initiation and progression of tumor by targeting multiple oncogenes, such as c-Met, FHL2, ROCK1, and SKP2 [11]. Importantly, numerous evidences showed that induced miR-340-5p expression promotes cellular apoptosis and suppresses cell viability, thus inhibiting tumor development [12–14]. Besides involving in tumorigenesis, miR-340-5p is also found to be upregulated in a human failing heart and regulates cardiotrophin-1 thus induces cardiac eccentric hypertrophy [15]. Significantly elevated miRNA-340 expression levels have also been found in whole blood cells from gestational diabetes [16], implying a potential relationship between miRNA-340 and diabetes. Although a recent study has identified a significant elevation of miRNA-340-5p in the hearts from db/db mice by small RNA sequencing in comparison with wt mice [9], it is still unclear whether increased miR-340-5p levels in the DM heart are associated with the pathological changes in DCM progression.

The present study found upregulation of miR-340-5p in the heart tissues of diabetic db/db mice as well as in cardiomyocytes under diabetic conditions. We demonstrated that miR-340-5p directly binds to Mcl-1, an antiapoptotic molecule, leading to enhanced oxidative stress and mitochondrial dysfunction, thereby increasing myocardial cell death. We also demonstrated that the treatment of recombinant adeno-associated virus of type-9 (rAAV9) containing miR-340-5p tough decoy (TUD) rescued cardiac dysfunction in diabetic db/db mice, which is a potential therapeutic target for DM-associated cardiac dysfunction.

## 2. Materials and Methods

### 2.1. Human Samples

Human plasma samples were collected at Sun Yat-sen Memorial Hospital (Guangzhou, China) between January 2020 and October 2021. The study was approved by the Ethics Review Board of Sun Yat-sen Memorial Hospital and was registered (SYSEC-KY-KS-2021-239), which conforms to the principles outlined in the Declaration of Helsinki. T2DM was defined according to the American Diabetes Association criteria as follows: a fasting plasma glucose level ≥ 7.0 mmol/l or 2 hours postprandial plasma glucose readings ≥ 11.1 mmol/l by multiple determinations. T2DM patients with heart failure (HF) were considered with symptomatic HF (New York Heart Association class II to IV) for at least 3 months and left ventricular ejection fraction (LVEF) < 40%. Patients with a history of acute coronary syndrome, primary valvular heart disease, atrial fibrillation, pulmonary heart disease, viral myocarditis, chronic viral or bacterial infections, tumors, or immune disorders were excluded. Detailed information of all the patients was obtained on demographics and clinical manifestation, and cardiac function was determined by echocardiography.

### 2.2. Animals and rAAV Injection

Leprdm/+ on a C57BL/6J background (db/db) and C57BL/6J (wt) mice were purchased from Biocytogen Co., Ltd. (Beijing, China). Age-matched mice were maintained on a 12-hour light-dark schedule with access to standard chow and water at the laboratory animal center of Guangzhou Medical University. The genotypes were determined by the PCR performed on tail DNA. The STZ-induced DM mouse model was performed by continuous intraperitoneal injection with 50 mg/kg STZ for 5 days as we described previously [17]. For induction of the DM rat model, 8-week-old male Wistar rats were given STZ by injection intravenously at a dose of 60 mg/kg. The rAAV9 system was purchased from GeneCopoeia, Inc. (Rockville, MD, USA). Plasmids for rAAV9 package as miR-random, miR-340-5p, or anti-miR-340-5p were obtained from Thermo Fisher Scientific Inc. (Waltham, MA, USA). The rAAV9 vectors were prepared by cotransfection with plasmid in human embryonic kidney 293 cells as described [18]. Male wt and db/db mice were randomly divided into different groups and injected with rAAV (1 × 10^11^ genome copies into 100 *μ*l saline per animal) via tail vein at 6 weeks old. Cardiac echocardiography and hemodynamics were performed after every 6 weeks of injection. At the end of the experiments, all the animals were fasting for 12 hours and plasma was collected for the measurement of glucose, insulin, triglycerides (TGs), and total cholesterol (TC) (Jiancheng Biotechnology, Nanjing, China). Then, the mice were euthanized by CO_2_ inhalation after cardiac function analysis. All the experimental protocols were conducted with approval of the Guidelines of Animal Care and Use Committee of Guangzhou Medical University.

### 2.3. Cardiac Echocardiography and Hemodynamics

Mice were exposed to the inhalation anesthetic isoflurane (1.5%~2%). M-mode and tissue Doppler interrogation were performed by a 30 MHz high-frequency scan head (Vevo770, VisualSonics, Toronto, Canada), and LV end-diastolic diameter (LVDd), LV end-systolic diameter (LVSd), percentage of fractional shortening (FS%), and the average ratios of *E*/*A* and *E*/*e*′ were measured as we performed previously [19].

For hemodynamic measurements of mice, anaesthesia was maintained by inhalation of 2% isoflurane and a 1.4-French high-fidelity micromanometer-tipped catheter (no. SPR-671 from Millar Instruments Inc. Houston, TX, USA) was inserted through the isolated right carotid artery into the ventricular. Hemodynamic parameters, including LV systolic pressure (LVSP), LV end-diastolic pressure (LVEDP), and rate of LV pressure rise (+dP/dt and −dP/dt), were measured both at baseline and in response to infusions of 10 ng isoproterenol.

### 2.4. Histopathology

Formalin-fixed heart tissues were embedded in paraffin and sectioned into 4 *μ*m slices. The morphological observation was determined by H&E staining. To detect the intestinal fibrosis, sections were stained with Sirius red. For cell surface area (CSA) measurement, the sections were stained with 50 *μ*g/ml wheat germ agglutinin (WGA, Sigma-Aldrich, St. Louis, MO, USA) as we described previously [19]. Tissue sections were then visualized by a microscope and photographed. The myofibril loss and fibrotic area were determined by Image-Pro Plus Version 6.0 (Media Cybernetics, Inc., Rockville, MD, USA).

### 2.5. Terminal Deoxynucleotidyl Transferase (TUNEL) Assays

The frozen sections of the heart from wt and db/db mice were cut into 10 *μ*m thickness and mounted. The apoptotic cells in the myocardium were determined by APO-BrdU TUNEL Assay Kit (A23210, Thermo Fisher Scientific Inc.) according to the manufacturer's instructions followed by fixing with 4% formaldehyde.

### 2.6. DHE Staining

Unfixed frozen heart sections were rinsed by phosphate-buffered saline (PBS) and immersed in the 10 mM dihydroethidium (DHE, Merck, Kenilworth, NJ, USA) staining solution in a 37-centigrade humid chamber for 30 min followed by rinse and fixation with 4% formaldehyde. Fluorescence of ethidium was observed with the confocal microscopy, and mean fluorescence intensity (MFI) was determined by Image-Pro Plus Version 6.0.

### 2.7. Total GSH Assay and SOD Activity Assay

The total concentrations of GSH and oxidized glutathione (GSSG) in heart tissue or cultured cells were determined with the Colorimetric GSH Assay Kit (ab239709, Abcam, Cambridge, UK) following the manufacturer's protocol. Total SOD activity of heart tissue homogenates or cell lysates was detected with Superoxide Dismutase Activity Assay Kit (ab65354, Abcam) according to the manufacturer's instructions.

### 2.8. Mitochondrial Function Analysis

The isolated mitochondrial in the myocardium was used to assess mitochondrial respiration. Briefly, after being perfused with 10 mM glucose and homogenized, cardiac mitochondria were isolated from the whole freshly harvested hearts by using Mitochondria Isolation Kit (ab110168, Abcam) following the manufacturer's protocol. The isolated mitochondria were then kept at 25 centigrade and used for further analysis. To measure mitochondrial respiration, isolated mitochondria were incubated in a respiration medium (3 mM HEPES, pH 7.4, 120 mM KCl, 5 mM KH_2_PO_4_, 1 mmol/l EGTA, 10 mM glucose, and 1 mg/ml BSA). The substrates of 2 mM malate, 5 mM L glutamate, and 10 mM pyruvate were added 2 hours before the assay, and the state 2 rate of respiration was measured. Following adenosine diphosphate (ADP; 1 mM, Merck Millipore, Burlington, MA, USA) addition, state 3 maximally stimulated respiration was measured. The state 4 rate of respiration rates in the absence of ADP phosphorylation was measured followed the addition of uncoupled regent 1 *μ*g/ml oligomycin. The total ATP contents of the samples were determined using Luminescent ATP Detection Assay Kit (ab113849, Abcam) following the manufacturer's instructions and the ATP synthesis, and ATP to O ratio was calculated as described [20]. For measurement of oxygen consumption rates (OCR) of HL-1 cells, a flow culture system was used that concomitantly measure OCR while collecting outflow fractions for subsequent measurement of lactate. OCR was calculated as the product of the flow rate (approximately 150 *μ*l/min) and the difference between inflow and outflow levels of oxygen per 10^6^ cells, which was measured by detecting the phosphorescence lifetime of an oxygen-sensitive dye (ab197242, Abcam).

To the analysis of mitochondrial oxidative phosphorylation (OXPHOS), the activities of complex I (NADH dehydrogenase, the first protein complex in the electron transport chain) and complex IV (cytochrome c oxidase, the final protein complex in the electron transport chain) were analyzed. The isolation and purification of mitochondria were performed as described above. The enzyme activity of complex I and IV was determined by a colorimetric analysis following the manufacturer's protocols, respectively (ab109721 and ab109911, Abcam).

### 2.9. Electron Microscopy

Heart tissue was removed and immersed in 0.5% glutaraldehyde and 0.1% tannic acid in cold PBS. After 4 hours, heart tissues were transferred to Karnovsky's fixative buffer on ice overnight. After cut into 1 mm^2^ pieces, the heart tissues were postfixed in 1% osmium tetroxide (pH 7.4) for 1 hour and washed by PBS. Then, samples were dehydrated and embedded in epoxy resin. Sections of heart tissues were cut into 0.2 *μ*m and stained with uranyl acetate and 0.2% lead citrate. Images were photographed under an H-7650 electron microscope (Hitachi, Tokyo, Japan).

### 2.10. Cardiomyocyte and Noncardiomyocyte Isolation and Fluorescence In Situ Hybridization (FISH)

The isolations of cardiomyocytes and noncardiomyocytes from wt and db/db mice were performed based on established procedures. The two hearts were pooled per collection and replicate at 4 independent experiments. The probe for miR-340 (microRNA probe, SpectrumGreen directly labeled, Creative Biolabs, Shirley, NY, USA) was add to the culture cells after incubated with proteinase K (2.0 nM, 10 min). The cell-type-specific markers troponin I and vimentin were costained with miR-340. For neonatal rat ventricular myocytes (NRVMs) and cardiac fibroblast preparation, a standard method was carried out as we described previously [19].

### 2.11. Cell Culture

The AC16, HL-1, and H9c2 cardiomyocyte lines were purchased from ATCC and cultured in modified Eagle's medium/Nutrient Mixture F-12 (MEM/F12) supplemented with 10% fetal calf serum (FCS), 4 mM glutamine, and penicillin (100 U/ml)/streptomycin (100 U/ml). The miR-340-5p mimic, miR-340-5p inhibitor, and negative control were obtained from GenePharma (Shanghai, China). To knockdown Mcl-1 in HL-1 cells, a mouse small interfering RNA (siRNA) reagent that targeted the Mcl-1 gene (sense 5′-GUAAGGACGAAG-CGGGACU-3′; anti-sense 5′-GUACUUGACUUAAGGUUCACU-3′, Thermo Fisher Scientific Inc.) was used. pcDNA 3.1(+) vectors containing Mcl-1 (Thermo Fisher Scientific Inc.) were used for transfection to induce overexpression. All the transfections of the miRNA, siRNA, and plasmid DNA into the HL-1 were carried out by using Lipofectamine 3000 reagent (Invitrogen) according to the manufacturer's protocol.

### 2.12. RNA Extraction and Quantitative RT-PCR

Total RNA was extracted by using TRIzol® Reagent (Invitrogen, Carlsbad, CA, USA) as described [21]. The cDNA was synthesized using the Oligo Reverse Transcriptase Kit (Roche, Basel, Switzerland) following the manufacturer's instructions. Real-time quantitative PCR was carried out with the Fast SYBR Green Master Mix (Applied Biosystems, Foster City, CA, USA) on an Applied Biosystems 7900HT Fast Real-Time PCR System (Applied Biosystems). For quantifying circulating miRNAs, we followed the early recommendation of using C. elegans miRNA (C. elegans-miR-39) as a selecting right internal control (miRB0000010, Ribobio, Guangzhou, China). The primer sequences used in this study were as follows:

miR-340-5p, 5 ′-GTCGTATCCAGTGCAGGGTCCGAGGTATTCGCACTGGATACGACAATCAG-3′; mouse nppa, forward: 5′-GCTTCCAGGCCATATTGGAG-3′; reverse 5′-GGGGGCATGACCTCATCTT-3′; mouse nppb, forward: 5′-GAGGTCACTCCTATCCTCTGG-3′; reverse 5′-GCCATTTCCTCCGACTTTTCTC-3′; mouse Col1a1, forward: 5′-ACGCCATCAAGGTCTACTGC-3′; reverse 5′-ACTCGAACGGGAATCCATCG-3′; mouse Col3a1, forward: 5′-CCCTGGACCTCAGGGTATCA-3′; reverse 5′-GGGTTTCCATCCCTTCCAGG-3′; mouse Mcl-1, forward: 5′-TGTAAGGACGAAACGGGACT-3′; reverse 5′-CAAAAGCCAGCAGCACATT-3′; mouse Bcl2L11, forward: 5′-GAGATACGGATTGCACAGGA-3′; reverse 5′-ATTTGAGGGTGGTCTTCAGC-3′; and mouse GAPDH forward: 5′-ACTCCACTCACGGCAAATTC-3′; reverse 5′-TCTCCATGGTGGTGAAGACA-3′.

The difference in expression was calculated using the 2^−*ΔΔ*Ct^ method.

### 2.13. Protein Extraction and Western Blotting

LV tissues were homogenized in sufficient volumes of RIPA Buffer (Thermo Fisher Scientific Inc.), supplemented with protease inhibitor cocktail (Roche). The supernatant was prepared for western blot, and equal amounts of proteins were boiled with loading buffer (Thermo Fisher Scientific Inc.) and subjected to SDS-PAGE. The proteins were subsequently transferred to polyvinylidene fluoride (PVDF) membranes. Primary antibodies used were as follows: anti-Glut1 (1 : 200, sc-7903, Santa Cruz Biotechnology, Inc., Dallas, Texas, USA.), anti-Glut4 (1 : 1000, ab216661, Abcam), anti-Bcl-2 (1 : 500, sc-7382, Santa Cruz Biotechnology, Inc.), anti-BAX (1 : 200, sc-20067, Santa Cruz Biotechnology, Inc.), anti-cleaved caspase-3 (1 : 1000, ab2302, Abcam), anti-Mcl-1 (1 : 2000, ab28147, Abcam), and anti-GAPDH (1 : 2000, MAB374, Merck Millipore). The secondary antibody used was horseradish peroxidase-conjugated, and the bands were viewed with the ImageQuant LAS 4000 system (GE Healthcare, Fairfield, CT, USA).

### 2.14. Dual-Luciferase Assay

To perform the dual-luciferase assay, the synthetic wild-type and mutant Mcl-1 mRNA 3′-UTR fragments with miR-340-5p combining site were first amplified and subcloned into the pmirGLO luciferase plasmid vector (Promega, Madison, WI, USA) as described previously [22]. The luciferase reporter plasmid was then cotransfected with miR-340-5p mimic into AC16, HL-1, and H9c2 cells by Lipofectamine 3000, respectively. After 24 hours of transfection, the luciferase activity was determined by a dual-luciferase assay kit (E1910, Promega) and luminance was detected by using SpectraMax i3x Microplate Reader (Labcompare, San Francisco, CA, USA).

### 2.15. RNA Pulldown Assay

The RNA pulldown assay was conducted following a published method [23]. Briefly, HL-1 cells were transfected with 50 mM 3′-biotinylated miR-340-5p mimic or random (Thermo Fisher Scientific Inc.) for 24 hours. The transfected cells were washed by PBS and lysed in a protease inhibitor containing RNase-free buffer (20 mM Tris-HCl, 100 mM KCl, 5 mM MgCl_2_, and 0.5% NP-40). The lysates were incubated with streptavidin-Dynabeads M-280 (Invitrogen) for 8 hours. Then, the beads were washed by DNase I (Thermo Fisher Scientific Inc.) and boiled in SDS buffer. After samples were collected by the miRNeasy Mini Kit (Thermo Fisher Scientific Inc.), the RNAs present in the pulldown material were detected by RT-PCR.

### 2.16. RIP-RT-PCR

HL-1 cells were transfected with miR-21 mimics for 24 hours and lysed and then immunoprecipitated with anti-Ago2 antibody (M00189, BosterBio, Pleasanton, CA, USA) or IgG using protein A/G magnetic beads (Thermo Fisher Scientific Inc.). After washed and resuspended, the remaining products were extracted with TRIzol, and the levels of mcl-1 and 18s mRNA were quantified by reverse transcription PCR.

### 2.17. MitoTracker Staining

Followed the treatment with high glucose and palmitate, HL-1 cells were washed twice with PBS and added prewarmed diluted staining solution containing 200 nM MitoTracker Red CMXRos (M7512, Thermo Fisher Scientific Inc.) for incubation of 45 minutes. Subsequently, the cells were fixed with 4% formaldehyde and photographed by an Olympus FV1000-D (BX) laser scanning confocal microscope (Olympus Life Science, Tokyo, Japan). The intensity of the MitoTracker was calculated by Image-Pro Plus Version 6.0 software.

### 2.18. Cell Apoptosis Detection

Cellular apoptosis assay was performed by flow cytometry as previously described [24]. HL-1 cells with different additional treatments were subsequently stained with Annexin V-FITC paired with 7-AAD (640922, BioLegend, San Diego, CA, USA). The percentage of cellular apoptosis was then determined using the FSCAN flow cytometer (BD Biosciences, Franklin Lakes, NJ, USA).

### 2.19. Statistical Analysis

The statistical calculations were performed by GraphPad Prism 8 Software (GraphPad Software Inc., San Diego, CA, USA). Clinic data are expressed as tabulated percentiles from 10 to 90. Other data are presented as the mean ± standard error of the mean (SEM). Differences between groups were analyzed by the Mann-Whitney test or unpaired two-tailed Student's *t*-test when comparison was determined in two groups. To compare multiple groups, comparison was determined with one-way analysis of variance followed by the Tukey-Kramer test. Correlation between miRNA-mRNA expression was conducted by Pearson correlation analysis. A value of *P* < 0.05 was considered significant differences.

## 3. Results

### 3.1. miR-340-5p Is Upregulated in Cardiomyocytes under Diabetic State

**Figure 1:**
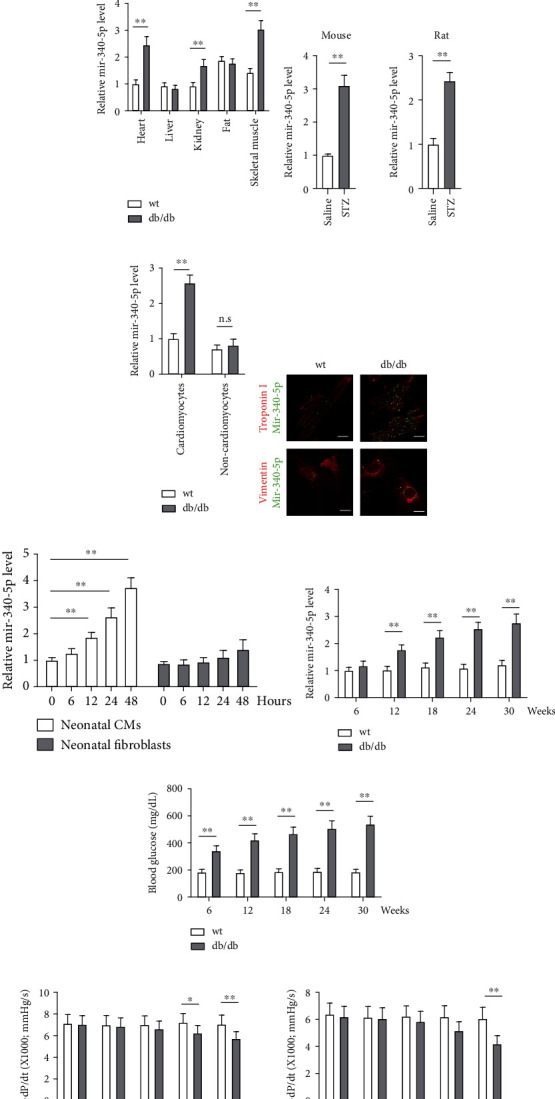
miR-340-5p is upregulated in cardiomyocytes of diabetic mice. (a) Quantitative reverse transcription polymerase chain reaction (qPCR) analysis of miR-340-5p expression in plasma of health control and diabetic patients (left) and DM patients without heart failure and DM patients with heart failure (right). (b, c) qPCR analysis of miR-340-5p expression in plasma (b) and various tissues (c) from wild-type (wt) and db/db mice at 30 weeks of age. miR-340-5p levels in other tissues of db/db mice are represented as fold changes relative to the heart. *n* = 10 in wt and db/db mice. (d) qPCR analysis of miR-340-5p expressions in heart tissues of STZ-induced DM in mice (left) and rats (right). *n* = 8 in each group of animals. (e) qPCR analysis of miR-340-5p in cardiomyocytes (CMs) and noncardiomyocytes (non-CMs) from db/db mice. *n* = 4 independent experiments. (f) Fluorescence in situ hybridization (FISH) analysis determined miR-340-5p expressions in cardiomyocytes (stained with troponin I) and cardiac fibroblasts (stained with vimentin). Scale bar: 5 *μ*m. (g) Time course analysis of miR-340-5p expressions in neonatal rat ventricular cardiomyocytes (NRVMs) and neonatal rat fibroblasts cultured with high glucose (30 mM) and palmitate (0.5 mM) at each time point (0, 6, 12, 24, and 48 hours after treatment). *n* = 4 at each time point. (h–j) Time course analysis of cardiac miR-340-5p expressions (h), fasting glucose (i), and cardiac function represented as +dP/dt (a measure of contractile function) and −dP/dt (a measure of the ability of the heart to relax, j). *n* = 6, 6, 7, 8, and 8 at each time point. Data were expressed as the tabulated percentiles from 10 to 90 (a) and the mean ± SEM (b–j). ^∗^*P* < 0.05 and ^∗∗^*P* < 0.01. n.s.: nonsignificant.

In order to reveal the association between miR-340-5p and DCM, we collected clinical serum samples from diabetic patient and age-matched health control (Table S1). We first determined that the expressions of miR-340-5p were upregulated in the patients with DM compared with the nondiabetic patients. Moreover, the expressions of miR-340-5p were much higher in the failing heart of patients with DM compared with diabetic patients without cardiac dysfunction (Figure 1(a), Table S2). Leptin receptor-deficient db/db mice are a widely used preclinical model in diabetes research. In a microarray analysis of the db/db mouse hearts compared to wt controls, miR-340-5p was observed notably upregulated among 21 dysregulated miRNAs [9]. In this study, we verified that miR-340-5p expressions were significantly elevated both in the plasma and heart tissues (Figures 1(b) and 1(c)) of db/db mice. We also observed higher levels of expression of miR-340-5p in the heart tissues of STZ-induced diabetic mice and rats than in those treated with saline (Figure 1(d)). These results indicated that high miR-340-5p expressions in diabetic rodents are obesity-independent hyperglycemic factors. By RT-qPCR, we found that miR-340-5p was specifically elevated in diabetic cardiomyocytes (Figure 1(e)). We further confirmed that miR-340-5p expression is comparable in diabetic and normal fibroblasts but much higher in cardiomyocytes in db/db mice by in situ hybridization (Figure 1(f)). The time-dependent expressions of miR-340-5p were also measured in rat neonatal cardiomyocytes and cardiac fibroblasts. miR-340-5p were increased in neonatal cardiomyocytes after 12 hours of treatment with high glucose (30 mmol/l) and palmitate (0.5 mmol/l), and it remained increased until the end of treatment. However, the expression in cardiac fibroblasts was comparable and showed a slight increase after culture for 48 hours (Figure 1(g)). In addition, we found that compared to wt, db/db mice presented higher blood glucose from the early age, which was preceded the elevation of cardiac miR-340-5p expression (Figures 1(h) and 1(i)), and both increases occurred prior to the observable cardiac dysfunction determined by hemodynamic analysis in db/db mice (Figure 1(j)), suggesting a possible causal role of the high-glucose-induced miR-340-5p expression in the progression of DCM.

### 3.2. Cardiac Dysfunction Is Exacerbated by miR-340-5p Overexpression and Rescued by miR-340-5p Inhibition in db/db Mice

**Figure 2:**
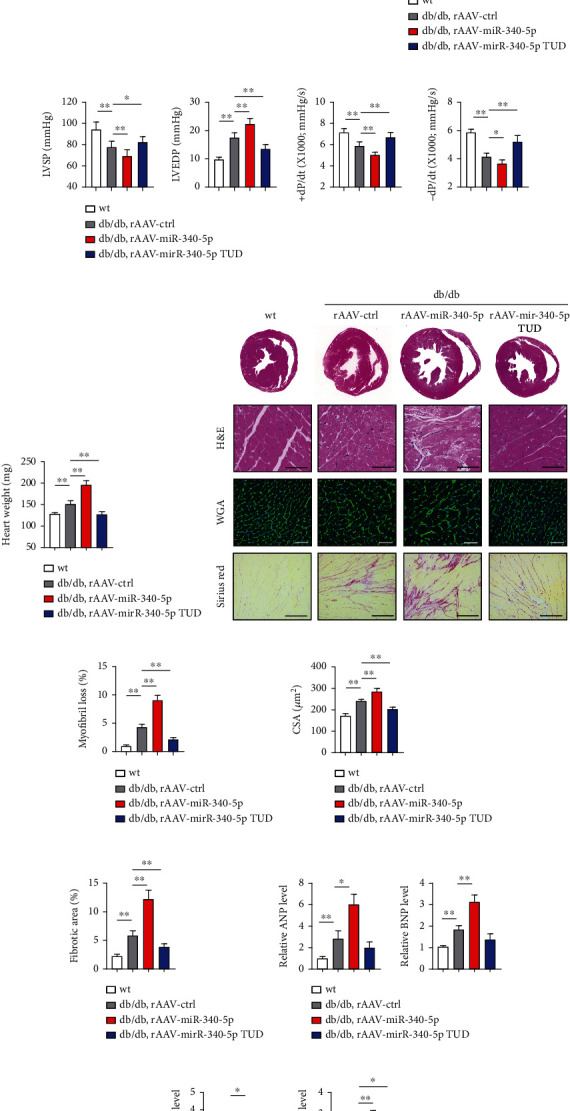
Cardiac dysfunction in DM is exacerbated by miR-340-5p overexpression. (a) Representative images and quantitative analysis of M-mode echocardiography and tissue Doppler examination of 30-week-old wt and db/db mice under rAAV-miR-340-5p or rAAV-miR-340-5p TUD treatment. Left ventricular end-diastolic diameter (LVDd), FS% (fractional shortening), E/A ratio, and E/e′ ratio. *n* = 6 − 8 in each group of mice. (b) Hemodynamic analysis of 30-week-old wt and db/db mice under different treatments. Left ventricular systolic pressure (LVSP), left ventricular end-diastolic pressure (LVEDP), +dP/dt, and −dP/dt were measured. *n* = 8 in each group of mice. (c) Heart weight was measured. *n* = 8 in each group of mice. (d) Representative images of morphologic staining of the hearts in wt and db/db mice with different treatments. H&E: scale bar: 100 *μ*m. WGA: scale bar: 100 *μ*m. Sirius red: scale bar: 200 *μ*m. (e–g) Quantitative analysis of myofibril loss (e), cell surface area (CSA, f), and fibrotic area (g) in wt and db/db mice with different treatments. *n* = 6 − 8 in each group of mice. (h, i) qPCR analysis of the hypertrophic mRNA markers, ANP and BNP (h), as well as the cardiac fibrosis markers, Col1a1 and Col3a1 (i). *n* = 8 in each group of mice. Data were represented as the mean ± SEM. ^∗^*P* < 0.05 and ^∗∗^*P* < 0.01.

To illustrate the causative role of miR-340-5p in DM-induced cardiac dysfunction, a rAAV9 system (with the TnT cardiac-specific promoter at 1 × 10^11^ vector copy numbers) was used to overexpress miR-340-5p or miR-340-5p inhibitor based on the TUD design and injected the vectors into 8-week-old db/db mice. The efficiency of rAAV delivery was determined by real-time PCR of miR-340-5p in the heart (Supplementary Figures 1(a) and 1(b)), and the body weight, blood glucose level, and insulin concentration did not show the difference among mice after rAAV delivery (Supplementary Figures 1(c)–1(e)). In addition, no difference was observed between the mice injected with rAAV-miR-340-5p or rAAV-miR-340-5p TUD in the distribution of triglycerides (TGs) and total cholesterol (TC) (Supplementary Figures 1(f) and 1(g)). Moreover, we also measured the Glut1 and Glut4 expression in wt and db/db mice after rAAV delivery. An approximate level of Glut1 and a reduced Glut4 was observed in heart tissues of db/db mice compared to those of wt mice. However, the expressions were comparable in db/db mice treated with either rAAV-miR-340-5p or rAAV-miR-340-5p TUD, suggesting that miR-340-5p might not affect glucose uptake in heart tissues (Supplementary Figure 1(h)). At 30 weeks of age, wt and db/db mice with different treatments were subjected to cardiac function measurements. Although no significant difference was observed between wt mice treated with rAAV-miR-340-5p or miR-340-5p inhibitor, severe left ventricular dilatation was found in miR-340-5p-overexpressed db/db mice, while miR-340-5p inhibition rescued ventricular dilatation. Besides worsening cardiac systolic dysfunction, diastolic dysfunction, which represents the earliest preclinical manifestation of DCM, was also found to be more deteriorative in db/db mice. On the other hand, mice treated with rAAV-miR-340-5p inhibition of miR-340-5p notably prevented the worsening of LVDd, FS%, *E*/*A*, and the *E*/*e*′ ratio in db/db mice (Figure 2(a)). To further illustrate the changes in systolic and diastolic functions, LVSP, LVEDP, +dP/dt, and -dP/dt were measured by invasive hemodynamic monitoring. These parameters were all significantly worse in db/db mice treated with rAAV-miR-340-5p and alleviated by miR-340-5p inhibitor treatment (Figure 2(b)). No significant difference was observed in wt mice treated with rAAV-miR-340-5p or rAAV-miR-340-5p TUD treatment (Supplementary Figures 2(a) and 2(b)). Therefore, these findings suggested that miR-340-5p exacerbates cardiac dysfunction in db/db mice.

Heart weights were measured and were found to be significantly increased in miR-340-5p-overexpressed db/db mice but alleviated by miR-340-5p inhibition (Figure 2(c)). Morphologic analyses using H&E, WGA, and Sirius red staining were also performed at 30 weeks of age for each group of mice (Figure 2(d)). Diabetes caused a focal, progressive loss of cardiac myofibrils, which was quantified and found further worsened in db/db mice after treatment with rAAV-miR-340-5p, while miR-340-5p inhibitor attenuated increased myofibril loss in diabetic mice (Figure 2(e)). miR-340-5p-overexpression and inhibition affected heart weight accompanied by the changes in WGA staining-determined cardiomyocyte size (Figure 2(f)). Moreover, cardiac fibrosis was found elevated by overexpression of miR-340-5p and attenuated by inhibition of miR-340-5p in diabetic conditions (Figure 2(g)). Finally, the mRNA expression levels of the hypertrophic markers, ANP and BNP, as well as the fibrosis markers including Col1a1 and Col3a1, showed similar changes in rAAV-treated db/db mice (Figures 2(h) and 2(i)). Collectively, these findings indicated that cardiac hypertrophy and heart failure are exacerbated in db/db mice miR-340-5p but rescued by miR-340-5p inhibition.

### 3.3. Apoptosis and Reactive Oxygen Species (ROS) Production Are Increased and the Mitochondrial Functions Are Decreased in miR-340-5p-Overexpressed db/db Mice

**Figure 3:**
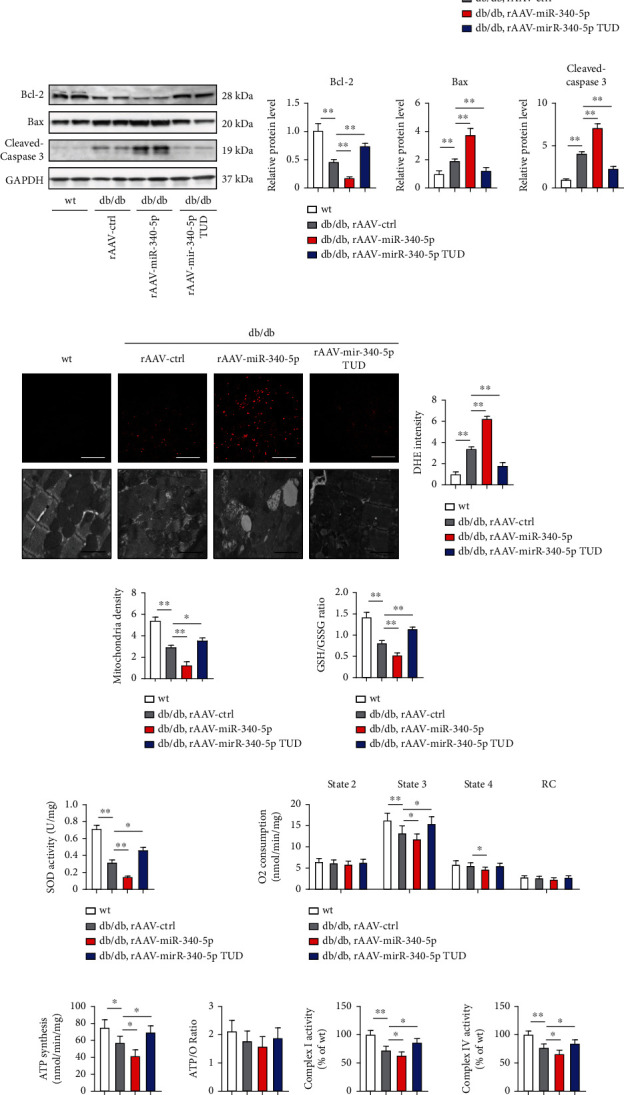
miR-340-5p overexpression enhances apoptosis, ROS production, and mitochondrial dysfunction in DM mice. (a) Representative images and quantitative analysis of terminal deoxynucleotidyl transferase dUTP nick end labeling- (TUNEL-) stained heart sections of wt and db/db mice under treatment with rAAV-miR-340-5p or rAAV-miR-340-5p TUD treatment. Scale bar: 100 *μ*m. *n* = 6 − 8 in each group. (b) Western blot analysis of Bcl-2, Bax, and cleaved caspase 3 expressions in heart tissues as well as quantification of each group of mice. *n* = 6 in each group. (c–e) Representative images of DHE staining (scale bar: 100 *μ*m) and electron microscopy (scale bar: 2 *μ*m) as well as quantification of DHE intensity (d, *n* = 6 in each group) and mitochondrial density (e, *n* = 6 in each group) of each group of mice. (f, g) Oxidative stress in the myocardial tissues was determined by measuring the glutathione (GSH) to oxidized glutathione (GSSG) ratio (f, *n* = 6 in each group) and superoxide dismutase (SOD, g, *n* = 6 in each group.) activity of each group of mice. (h–j) Mitochondrial function in the myocardial tissues was determined by measuring mitochondrial respiration rates (h, *n* = 4 in each group), the ATP synthesis rates (i, *n* = 6 in each group), and mitochondrial complex I and IV (j, *n* = 6 in each group) activity in isolated mitochondria of heart tissue in each group of mice. Data were represented as the mean ± SEM. ^∗^*P* < 0.05 and ^∗∗^*P* < 0.01.

Many studies have demonstrated that hyperglycemia-induced cardiomyocyte apoptosis leads to cell loss to decrease cardiac function in DCM [25, 26]. Thus, we used the TUNEL assay to identify apoptosis in the myocardium. A slight increase in TUNEL-positive cells was found in wt mice with cardiac miR-340-5p overexpression (Supplementary Figure 2(c)). However, a significant increase was observed in db/db mice with miR-340-5p overexpression, and miR-340-5p TUD inhibition attenuated apoptosis (Figure 3(a)). Levels of the two main proteins of the Bcl-2 family, namely, antiapoptotic Bcl-2 and proapoptotic proteins Bax, were determined by western blot analyses. Though there is no significant change in wt heart tissues (Supplementary Figure 2(d)), we found that expression of Bcl-2 was significantly decreased in the diabetic hearts transfected with miR-340-5p and this decrease was reversed in the miR-340-5p TUD-treated hearts, whereas Bax protein levels showed the reverse trend. Caspase-3 is one of the major effectors of apoptosis, and activation of caspase-3 (the cleaved form) is known to be accompanied by apoptosis. The expression of cleaved caspase-3 was also notably elevated in db/db mice treated with rAAV-miR-340-5p, but decreased in mice transfected with miR-340-5p TUD (Figure 3(b)). These data demonstrated that miR-340-5p exacerbates apoptosis in DCM. Apoptotic death is generally induced by oxidative stress, and mitochondria have a central role in cell death [27, 28]. Thus, we next analyzed the level of oxidative stress and mitochondrial function after miR-340-5p overexpression and inhibition. The heart sections of each group of mice were stained with DHE (Figure 3(c)) and found that the intensity of DHE was much more elevated in miR-340-5p-overexpressed db/db mice and decreased after miR-340-5p inhibition (Figure 3(d)). Mitochondrial morphology was observed by electron microscopy. In db/db heart tissues, disrupted cristae structures, mitochondrial vacuoles, and reduced mitochondrial density were observed, indicating the imbalance of mitochondrial function, whereas miR-340-5p TUD transfection reverses the abnormality of mitochondria (Figure 3(e)). Corresponding with the increased DHE intensity, the GSH to GSSG ratio and SOD activity were decreased in heart tissues of miR-340-5p-overexpressing and elevated in miR-340-5p inhibition db/db mice (Figures 3(f) and 3(g) and Supplementary Figures 2(e) and (f)). Mitochondrial respiration and ATP synthesis rates were measured to test the hypothesis that miR-340-5p-related mitochondrial dysfunction contributed to DCM. We isolated mitochondria in heart tissues from each group of mice. Basal respiration (state 2) was comparable in each group, while maximal ADP-stimulated mitochondrial oxygen consumption (state 3) was suppressed in db/db mice and decreased further in mice with miR-340-5p transfection and this decrease was reversed by miR-340-5p inhibition. State 4 mitochondrial respirations were also decreased in miR-340-5p-overexpressed db/db mice (Figure 3(h)). Moreover, mitochondrial ATP synthesis was decreased in db/db mice transfected with rAAV-miR-340-5p, whereas elevated by the treatment of miR-340-5p TUD. Meanwhile, no significant difference was observed in the ATP/O ratios (Figure 3(i)), which might be attributed to the fact that ATP and oxygen consumption rates were reduced proportionally. Mitochondrial dysfunction was also evidenced by decreased mitochondrial respiratory complex I and IV activity leading to reduced ATP synthesis in diabetic heart tissue, thus showed similar changes in db/db mice with different treatment (Figure 3(j)). Taken together, these findings demonstrated that miR-340-5p-overexpressed db/db mice show increased apoptosis and ROS production as well as decreased mitochondrial function compared to those treated with the rAAV control.

### 3.4. miR-340-5p Directly Targets Mcl-1 in Cardiomyocytes

**Figure 4:**
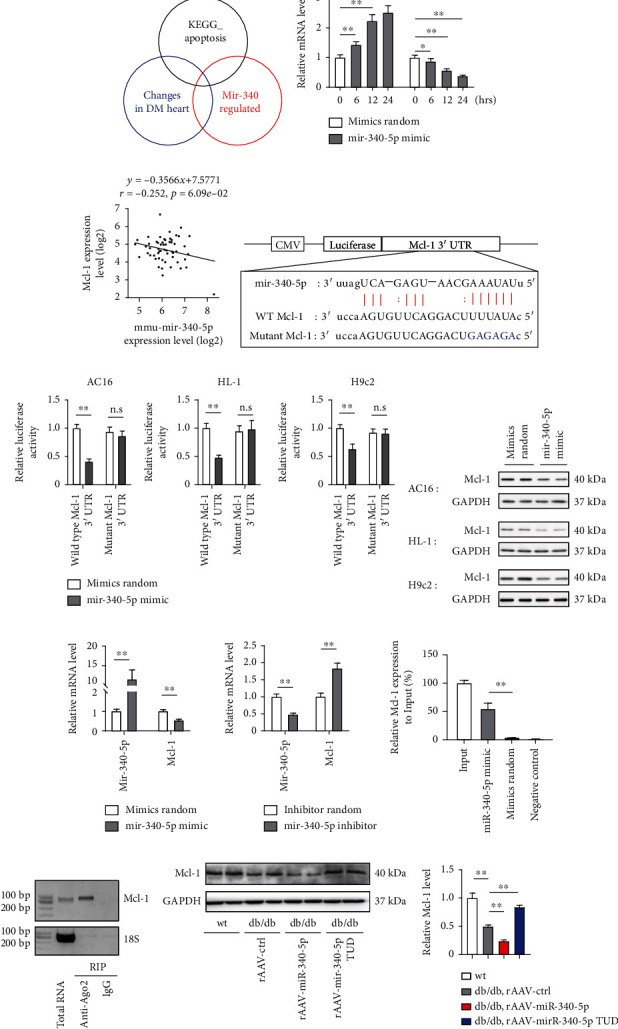
miR-340-5p directly targets the Mcl-1 apoptotic regulator. (a) Strategy to identify nuclear targets for miR-340-5p in diabetic cardiomyopathy. (b) Time course analysis of miR-340-5p and Mcl-1 expression in HL-1 cells cultured with high glucose (30 mM) and palmitate (0.5 mM) at each time point (0, 3, 6, and 12 hours after treatment), *n* = 4 at each time point. (c) Pearson correlation analyses between miR-340-5p and Mcl-1 expression were performed in heart tissues of db/db mice. Total 54 samples were measured. (d, e). Bioinformatics analysis showed that Mcl-1 was a target of miR-340-5p (d), and the luciferase activity was analyzed (e) using AC-16, HL-1, and H9c2 cells treated with either miR-340 mimic. *n* = 4 independent experiments. (f) AC-16, HL-1, and H9c2 cells were treated with miR-340 mimic for 24 hours, and western blot was used to measure Mcl-1 expression. *n* = 4 independent experiments. (g) HL-1 cells were treated with miR-340 mimic or miR-340-5p inhibitor, and qPCR was performed to detected mcl-1 mRNA expression. *n* = 4 independent experiments. (h, i) RNA pulldown (h) and RIP RT-PCR assay (i) were performed to confirm whether miR-340-5p directly target mcl-1 in HL-1 cells. *n* = 3 independent experiments. (j) Western blot analysis of Mcl-1 expression in wt and db/db mice under rAAV-random or rAAV-miR-340-5p treatment. *n* = 6 in each group of mice. Data were represented as the mean ± SEM. ^∗^*P* < 0.05 and ^∗∗^*P* < 0.01. n.s.: nonsignificant.

Previous studies have revealed that miRNAs interact with their target mRNAs at specific sites to induce translational repression or mRNA deadenylation [6]. StarBase v.3.0 (http://starbase.sysu.edu.cn/index.php) was used to verify potential miR-340-5p targeting (base-pairing of at least 7 consecutive nucleotides (allowing G:U wobbles)) [29]. Corresponding with the fact that miR-340-5p overexpression increased cellular apoptosis in the heart tissue of diabetic mice, miR-340-5p has also been indicated as a suppressor for cancer cell proliferation and metastasis by enhancing their apoptosis [12, 13, 30]. Thus we hypothesized that candidate genes representing proposed targets of miR-340-5p would be accompanied by genes dysregulated in cardiomyocytes in DM and in the KEGG apoptosis pathway (https://www.genome.jp/pathway/hsa04210) (Figure 4(a)) [31]. Under the strict screening conditions, we identified potential miR-340 target genes by bioinformatics analysis using the BiBiServ Tool, and 2 genes including Mcl-1 and Bcl2L11 were found (Table S3). In HL-1 cells under the exposure to high concentrations of glucose and palmitate, the expression of miR-340-5p was increased in a time-dependent manner, which was similar to the NRVMs shown in Figure 1(e). In contrast, the expression of Mcl-1 was decreased, which was accompanied by an increase in miR-340-5p (Figure 4(b)). However, Bcl2L11 expression was increased under diabetic conditions (Supplementary Figure 3), indicating that Mcl-1 might be a potential target of miR-340-5p when DCM occurs. We further determined that miR-340-5p expression displayed a significant negative correlation with Mcl-1 in heart tissues of db/db mice (Figure 4(c)). By using the miRNA-mRNA Interactome tool to predict the potential interaction position of Mcl-1 and miR-340-5p (Figure 4(d)), we next performed a dual-luciferase assay to certify whether miR-340-5p directly bind to Mcl-1 message. The constructed Mcl-1 fragment was subcloned downstream of the luciferase reporter gene, and the luciferase reporter gene was then cotransfected with miR-340-5p mimic into AC16, HL-1, or H9c2 cells. The wild-type 3′-UTR of Mcl-1 exhibited a reduced translation level in the presence of the miR-340-5p mimic, and similar results were observed in cardiomyocytes of different species. In addition, the mutated 3′-UTR failed to show the significant response to miR-340-5p (Figure 4(e)). AC16, HL-1, and H9c2 cells treated with the miR-340-5p mimic for 48 hours showed a significant reduction in Mcl-1 expression (Figure 4(f)), whereas miR-340-5p inhibitor treatment increased Mcl-1 mRNA expression levels (Figure 4(g)). Furthermore, RNA pulldown assay was conducted, and Mcl-1 was detected to be significantly higher in the miR-340-5p mimic group than in the random mimic group (Figure 4(h)). The RIP-PCR assay also showed that mcl-1 mRNA was detected by Ago2-RIP in miR-340-5p mimic-treated HL-1 cells (Figure 4(i)). Finally, upon rAAV-miR-340 treatment, Mcl-1 expression was reduced to an even greater extent in diabetic mice (Figure 4(j)). These data suggested that miR-340-5p directly targets Mcl-1.

### 3.5. miR-340-5p Induces Apoptosis by Targeting Mcl-1

**Figure 5:**
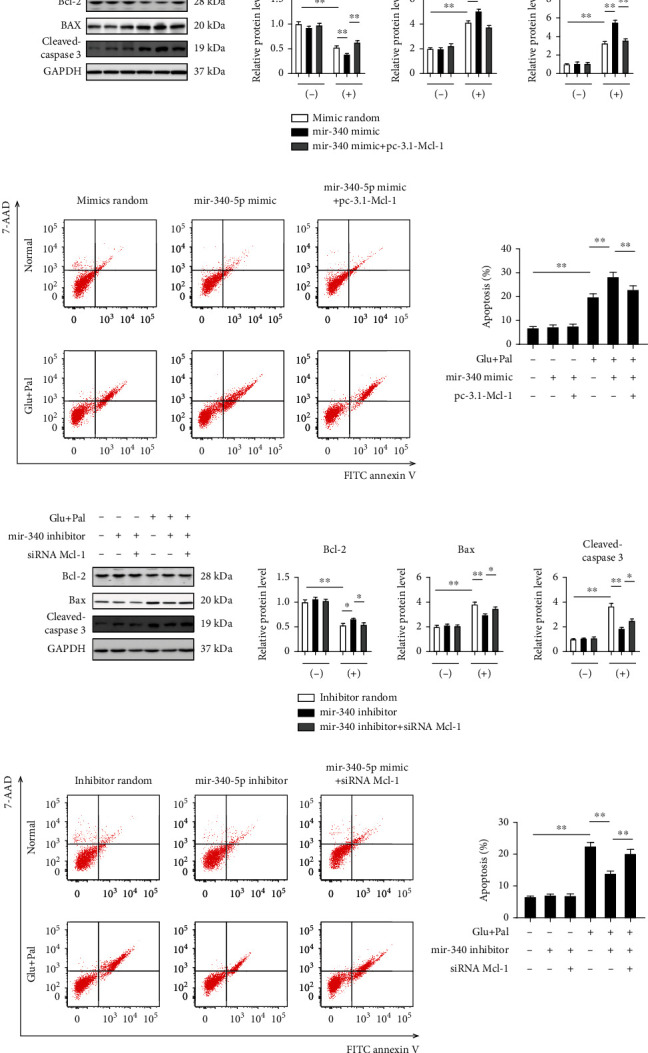
miR-340-5p regulates apoptosis by targeting Mcl-1. (a) Western blot analysis and quantification of Bcl-2, Bax, and cleaved caspase 3 expression in HL-1 cells under control or high glucose (30 mM)/palmitate (0.5 mM) conditions after transfection with random mimics, miR-340 mimic with or without pcDNA 3.1(+)-Mcl-1 plasmid for 48 hours. *n* = 4 independent experiments. (b) The apoptosis was measured by flow cytometry in HL-1 cells treated under diabetic condition for 6 hours after transfection with random mimics, miR-340 mimic with or without pcDNA 3.1(+)-Mcl-1 plasmid. *n* = 4 independent experiments. (c) HL-1 cells were cultured under control or high glucose (30 mM)/palmitate (0.5 mM) conditions after transfection with the miR-340 inhibitor with or without the siRNA mcl-1 for 48 hours. (d) The apoptosis was measured in HL-1 cells under the different conditions. *n* = 4 independent experiments. Data were represented as the mean ± SEM. ^∗^*P* < 0.05 and ^∗∗^*P* < 0.01.

To further elucidate the potential mechanisms between miR-340-5p/Mcl-1 and cellular abnormalities in diabetic conditions, we cultured HL-1 cells in the presence of high glucose and palmitate for 24 hours after transfection with miR-340-5p mimic or miR-340-5p inhibitor. Consistent with the effects of miR-340-5p, knocking down mcl-1 decreased antiapoptotic marker Bcl-2, but increased proapoptotic marker Bax and cleaved caspase 3 expressions, thus elevated cellular apoptosis in high glucose and palmitate-treated cells, while these results were reversed in mcl-1 overexpression HL-1 cells (Supplementary Figures 4(a) and 4(b)). In HL-1 cells treated with miR-340-5p mimic, Bcl-2 expression was decreased while Bax and cleaved caspase 3 levels were greatly elevated in diabetic state (Figure 5(a)), which resulted in increased apoptosis determined by flow cytometry (Figure 5(b)), while this increase was attenuated by knocking down mcl-1. On the other hand, the miR-340-5p inhibitor elevated the expression of Bcl-2, and high glucose and palmitate induced elevated Bax and cleaved caspase 3 levels were attenuated by the miR-340-5p inhibitor, but these effects were reversed by mcl-1 overexpression (Figure 5(c)). These results were also accompanied with cellular apoptosis measurement (Figure 5(d)). Taken together, it is suggested that miR-340-5p induces apoptosis by targeting mcl-1.

### 3.6. miR-340-5p/Mcl-1 Regulates Oxidative Stress and Mitochondrial Function in Diabetic Condition

**Figure 6:**
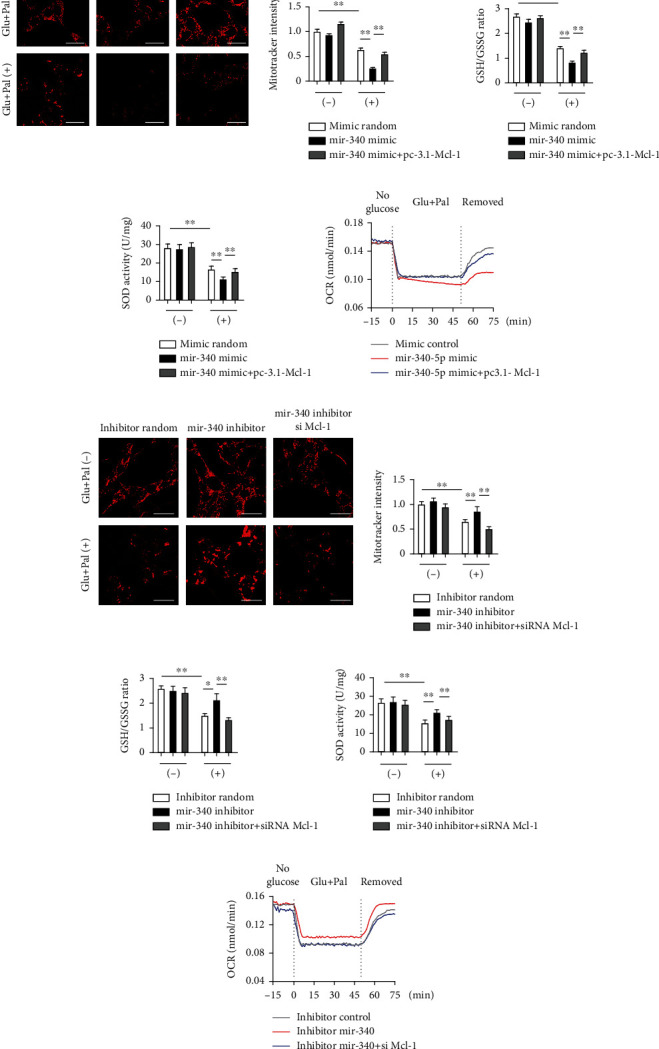
miR-340-5p/Mcl-1 regulates oxidative stress and mitochondrial function in diabetic condition. (a–c) HL-1 cells were cultured under control or high glucose (30 mM)/palmitate (0.5 mM) conditions after transfection with miR-340 inhibitor with or without Mcl-1 siRNA for 48 hours. MitoTracker staining and intensity analysis (a, scale bar: 10 *μ*m), GSH to GSSG ratio (b), and SOD activity (c) were determined. *n* = 4 independent experiments. (d) Temporal changes of OCR in HL-1 cells transfected with miR-340 mimic with or without pcDNA 3.1(+)-Mcl-1 plasmid in response to the following: step 1 (-15 to 0 min), no glucose; step 2 (0 to 55 min), addition of high glucose/palmitate; and step 3, removal of high glucose and palmitate (55 to 75 min). Data points are the average of two data sets controlled in parallel. (e–g) MitoTracker staining and intensity analysis (e, scale bar: 10 *μ*m), GSH to GSSG ratio (f), and SOD activity (g) were determined in HL-1 cells under different conditions. *n* = 4 independent experiments. (h) Temporal changes of OCR in HL-1 cells transfected with miR-340 inhibitor with or without Mcl-1 siRNA in response to addition and removal of high glucose/palmitate. The glucose and palmitate were then washed out as indicated. Data points are the average of two data sets controlled in parallel. Data were represented as the mean ± SEM. ^∗^*P* < 0.05 and ^∗∗^*P* < 0.01.

To determine the relationship between miR-340-5p and mitochondrial function, we measured the fluorescence intensity by MitoTracker Red assay, indicating the mitochondrial mass. Downregulation of Mcl-1 by siRNA decreased MitoTracker intensity and increased oxidative stress in high glucose and palmitate-treated cells, and the protective effects of Mcl-1 overexpression were also confirmed (Supplementary Figures 4(c)–4(e)). The MitoTracker intensity was decreased remarkably by the miR-340-5p mimic, which was rescued in Mcl-1-overexpressed HL-1 cells (Figure 6(a)). The miR-340 mimic treatment induced oxidative stress, which was measured by the cellular GSH to GSSG ratio and SOD activity (Figures 6(b) and 6(c)). Overexpression of Mcl-1 also reversed the miR-340-5p-induced repression of mitochondrial ATP synthesis and complex I and IV activity (Supplementary Figure 5(a)). Next, we detected the step changes of OCR in response to the diabetic condition. Reduction in OCR induced by high glucose and palmitate was significant in miR-340-5p mimic-treated HL-1 cells, and the recovery of OCR was limited after diabetic condition was removed. The impairment of OCR was rescued by Mcl-1 overexpression (Figure 6(d)). These data suggested that the miR-340-5p-induced loss of mitochondrial mass and function in diabetic conditions partly occurs through downregulated Mcl-1. When miR-340-5p was inhibited, the loss of mitochondrial mass and function under high glucose and palmitate treatment was alleviated, but this effect was prevented by knocking down Mcl-1 (Figure 6(e)). Increased cellular oxidative stress (Figures 6(f) and 6(g)) and decreased mitochondrial ATP synthesis and complex I and IV activity (Supplementary Figure 5(b)) occurred when Mcl-1 was knocked down in miR-340-5p-inhibited cells. Moreover, the OCR measurement indicated that miR-340-5p inhibition attenuated mitochondrial function in diabetic condition, while blocked by knocking down mcl-1(Figure 6(h)). These data suggested that miR-340-5p targeting Mcl-1 plays an indispensable role in regulating oxidative stress and mitochondrial function in the diabetic state.

## 4. Discussion

In the present study, we revealed that miR-340-5p targeting Mcl-1 is involved in DCM by regulating cardiac oxidative stress, mitochondrial function, and apoptosis. The miR-340-5p expression level was dramatically increased in the diabetic heart, and miR-340-5p overexpression directly led to the suppression of Mcl-1, which belongs to the Bcl-2 family and acts as an antiapoptotic protein. In turn, miR-340-5p overexpression contributed to several abnormalities in the heart, including aggravated mitochondrial loss and dysfunction, increased oxidative stress, increased ROS production, defective ATP production, enhanced myocardial apoptosis, and subsequent heart failure. Moreover, we showed that miR-340-5p inhibition effectively rescued cardiac dysfunction in diabetic mice, indicating the protective role of targeting miR-340-5p/Mcl-1 in regulating cardiac dysfunction in DCM.

DCM commonly has a long asymptomatic latent period associated with ventricular enlargement, fibrosis, abnormalities in cell signaling, and contractile dysfunction [3]. Although miR-340 is increased in gestational diabetic patients [16], the role of miR-340 in diabetes and diabetic complications remains unclear. In the present study, the expression of miR-340-5p was found increased in the heart much earlier than the cardiac dysfunction was observed, indicating that miR-340-5p may be the causative factor but not the result of DCM. miR-340-5p-overexpressed mice presented aggravated heart weight increase, myofibril loss, and fibrosis, which contributed to more serious dysfunction in db/db mice. Our results did not show direct evidence that miR-340-5p regulates increased fibrosis in the myocardium, but this increase may be the result of myocardium ROS production and profibrotic gene expression [32]. Interestingly, a recent study indicated that overexpression of miR-340-5p in human lung fibroblasts exerts a pulmonary antifibrotic effect by targeting TGF-*β*/p38/ATF1, thus inhibiting the activation and proliferation of fibroblasts [33]. These findings suggest that miR-340-5p is another cell-type-specific regulator and may present a reversal effect in different cell types, thus requiring further studies.

It is widely recognized that hyperglycemia-induced oxidative stress is a critical contributor to DCM in diabetic complications [3]. Increased oxidative stress refers to elevated intracellular levels of ROS, which are mainly generated inside mitochondria and can oxidize proteins, lipids, and DNA. Many studies showed that high ROS production induced by high glucose underlies different hyperglycemia-induced mechanisms, decreases the antioxidant capacity of the diabetic myocardium, and results in downstream events, including inflammation, hypertrophic changes, fibrosis, cardiomyocyte apoptosis, and contractile dysfunction [34–36]. Several types of miRNAs have been demonstrated to be involved in regulating ROS production in the heart, including miR-21, miR-34a, miR-144, miR-181, and miR-421 [10, 37–40]. Our results suggested that miR-340-5p might be another novel positive regulator of oxidative stress as supported by excessive ROS formation caused by cumulative damage to mitochondria and accompanied by exacerbated mitochondrial dysfunction, resulting in increased apoptosis and eventually contributing to the development of DCM in miR-340-5p-overexpressed diabetic mice. Despite the fact that the crucial role of mitochondria in ROS generation has been demonstrated, altered cardiomyocyte mitochondrial morphology in diabetic patients, including mitochondrial fragmentation, can be reversed by antioxidants, indicating that alterations in mitochondrial morphology and ROS production may reciprocally affect each other [41]. Altered mitochondrial function may also lead to metabolic changes, including decreased glucose and fatty acid utilization as well as increased myocyte lipid and diacylglycerol accumulation [27, 28]. Although we found there was no difference in lipid contents in plasma after miR-340-5p overexpression, additional details of miR-340-5p from cardiac metabolic analysis should be further considered.

Mcl-1 is an essential Bcl-2 family member that is not only upregulated in several kinds of human cancers but also highly expressed in the myocardium [42, 43]. Previous studies have found that cardiomyocyte-specific Mcl-1 knockout mice lead to rapid dilated cardiomyopathy and death [42]. Abnormal mitochondrial ultrastructure, including cristae disruption and swelling, and defective mitochondrial respiration are found in Mcl-1 knockout heart tissues, suggesting that Mcl-1 is essential for mitochondrial morphology and function [43]. Our results indicated that Mcl-1 expression was inhibited by miR-340-5p overexpression and thus might be responsible for mitochondrial loss and dysfunction in our diabetic model, while overexpression of Mcl-1 reversed miR-340-5p mimic-induced mitochondrial loss. Although it is still controversial, Mcl-1 has been suggested to be involved in the unique ability to prevent ROS production by inhibiting the upregulation of prooxidants through NOX4, which functions as a mitochondrial energetic sensor [44]. As an antiapoptotic protein, Mcl-1 deletion has also been found to dramatically elevate cellular apoptosis by acting as an inhibitor of Bax/Bak, the core regulators of the intrinsic pathway of apoptosis [43]. In addition, Mcl-1 knockdown reverses the prevention of ROS production and apoptosis associated with miR-340-5p inhibition. In line with these findings, our results indicated that cardiac Mcl-1 was significantly decreased in DCM, which may be partially responsible for the mitochondrial dysfunction, oxidative stress, and apoptosis related to miR-340-5p overexpression.

In the current study, we demonstrated for the first time that the miR-340-5p/Mcl-1 pathway induces cardiac oxidative stress and apoptosis in diabetic mice. The increase in cardiac expression of miR-340-5p under the diabetic state has also been disclosed in other studies [9]. Moreover, miR-340-5p is increased both in the failing human hearts and in a rat HF model induced by abdominal aorta-inferior vena cava fistula overload, indicating that miR-340-5p may affect the process of cardiac dysfunction [15]. In current study, miR-340-5p was verified to target mcl-1 and inhibits its expression in human, mouse, and rat cardiomyocytes, indicating that the sequences of mcl-1 might not highly conserved among these species. Though we identified mcl-1 as an important target of miR-340-5p in diabetic cardiomyopathy, there may be still other targets of miR-340-5p. In addition to our findings, miR-340-5p has also been found remarkably increased in a STZ-induced rat model of diabetic osteoporosis and downregulated runt-related transcription factor-2 (RUNX2) thus suppressed the bone formation [45]. Thus, these findings collectively support a negative role of miR-340-5p in the chronic complication of diabetes. Accumulating evidence suggests that miR-340-5p increases the expression levels of Bim, Bax, and cleaved caspase 3 but decreases the expression of Bcl-2, thereby contributing to increased apoptosis [12, 13, 30]. However, another study has reported that microRNA-340-5p plays a protective role in hypoxia/reoxygenation-induced apoptosis in an acute myocardial infarction model [46]. The difference in the response period for the animal model should be considered because the development of cardiomyopathy is chronic and progressive. Our results demonstrated that the decrease in cardiac diastolic function was much slower than the plasma glucose elevation in db/db mice. Importantly, we showed the evidence that TUD-based miR-340-5p inhibition was effective to attenuate ROS production and apoptosis in the myocardium and rescue cardiac dysfunction in diabetic mice, supporting the harmful role of miR-340-5p in the development of DCM.

## 5. Conclusions

Our findings revealed that cardiac abnormalities in db/db mice are closely associated with miR-340-5p overexpression through targeting Mcl-1 and aggravation of mitochondrial dysfunction and oxidative stress, thus increasing apoptosis. These findings suggested that targeting miR-340-5p might be a promising therapeutic strategy for the prevention of DCM.

## Data Availability

All data used to support the findings within the article are available from the corresponding author.

## Supplementary Materials

Supplementary Materials Supplementary Figure 1: miR-340-5p overexpression did not affect blood glucose or lipid profile in diabetic mice. (a) Sequence of rAAV-miR-340-5p inhibitor in TUD vector. (b) qRT-PCR analysis of miR-340-5p levels in heart tissues of mice at 30 weeks old (*n* = 8 in each group). (c) Body weight in wt and db/db mice transfected with different rAAV9 vectors (*n* = 8 in each group). (d–g) Fasting blood glucose (d), plasma insulin concentration (e), triglycerides (f), and total cholesterol (g) were measured in wt and db/db mice transfected with different rAAV9 vectors (*n* = 8 in each group). (h) Western blot analysis and quantification of Glut 1 and Glut 4 expression in heart tissues as well as quantification of each group of mice. *n* = 6 in each group. Data were represented as the mean ± SEM. Comparison determined with one-way ANOVA followed by the Tukey-Kramer test. ^∗^*P* < 0.01. Supplementary Figure 2: measurement of cardiac function and apoptosis in heart tissues of wt mice. (a) M-mode echocardiography examination of 30-week-old wt under rAAV-miR-340-5p or rAAV-miR-340-5p TUD treatment. Left ventricular end-diastolic diameter (LVDd), FS% (fractional shortening), *E*/*A* ratio, and *E*/*e*′ ratio. *n* = 6 in each group of mice. (b) Hemodynamic analysis of 30-week-old wt mice under different treatments. Left ventricular systolic pressure (LVSP), left ventricular end-diastolic pressure (LVEDP), +dP/dt, and −dP/dt were measured. *n* = 6 in each group of mice. (c) Representative images and quantitative analysis of TUNEL-stained heart sections of wt mice. Scale bar: 100 *μ*m. *n* = 5 − 6 in each group. (d) Western blot analysis of Bcl-2, Bax, and cleaved caspase 3 expressions in heart tissues as well as quantification of each group of mice. *n* = 6 in each group. (e, f) Oxidative stress in the myocardial tissues was determined by measuring the glutathione (GSH) to oxidized glutathione (GSSG) ratio and superoxide dismutase (SOD) activity of each group of mice. *n* = 6 in each group. Data were represented as the mean ± SEM. Comparison determined with one-way ANOVA followed by the Tukey-Kramer test. ^∗^*P* < 0.005. Supplementary Figure 3: increased Bcl2L11 expression in diabetic condition. Time course analysis of expression of miR-340-5p and Bcl2L11 in HL-1 cells treated with high glucose (30 mmol/l) and palmitate (0.5 mmol/l); the expression levels were determined by qRT-PCR (*n* = 4 independent experiments). Data were represented as the mean ± SEM. Comparison determined with one-way ANOVA followed by the Tukey-Kramer test. Supplementary Figure 4: Mcl-1 alleviates diabetic condition-induced apoptosis and mitochondrial injury. (a) Western blot analysis of Mcl-1, Bcl-2, Bax, and cleaved caspase 3 expressions in treated HL-1 cells under control or high glucose (30 mM)/palmitate (0.5 mM) conditions. (b) The apoptosis was measured by flow cytometry in HL-1 cells treated under diabetic condition for 6 hours after different treatments. (c–e) HL-1 cells were cultured in different treatment for 48 hours. MitoTracker staining and intensity analysis (c, scale bar: 10 *μ*m), GSH to GSSG ratio (d), and SOD activity (e) were determined. *n* = 4 independent experiments. Data were represented as the mean ± SEM. Comparison determined with one-way ANOVA followed by the Tukey-Kramer test. Supplementary Figure 5: miR-340-5p regulates mitochondrial dysfunction by targeting Mcl-1. (a) HL-1 cells were cultured under control or high glucose (30 mM)/palmitate (0.5 mM) conditions after transfection with the miR-340 mimic with or without the pcDNA 3.1(+)-Mcl-1 plasmid. Complex I activity and complex IV activity were measured and presented as percentage of control (*n* = 4 independent experiments). (b) HL-1 cells were cultured under control or high glucose (30 mM)/palmitate (0.5 mM) conditions after transfection with the miR-340 inhibitor with or without the siRNA Mcl-1. Complex I activity and complex IV activity were measured and presented as percentage of control (*n* = 4 independent experiments). Data were represented as the mean ± SEM. Comparison determined with one-way ANOVA followed by the Tukey-Kramer test. Table S1: clinic characteristics of patients in this study. Table S2: changed genes in a diabetic heart compared with control and filtered according to the predicted miR-340-5p target interactions. Table S3: changed genes in a diabetic heart compared with control and filtered according to the predicted miR-340-5p target interactions. Full scans of western blotting data are shown in figures. Full scans of western blotting data are shown in supplementary figures.
