# Supplementary material for: miR-340-5p Mediates Cardiomyocyte Oxidative Stress in Diabetes-Induced Cardiac Dysfunction by Targeting Mcl-1
Source: Oxid Med Cell Longev. 2022 Jan 27;2022:3182931. doi: 10.1155/2022/3182931 (PMC8813269; doi:10.1155/2022/3182931)

Supplementary figure 1

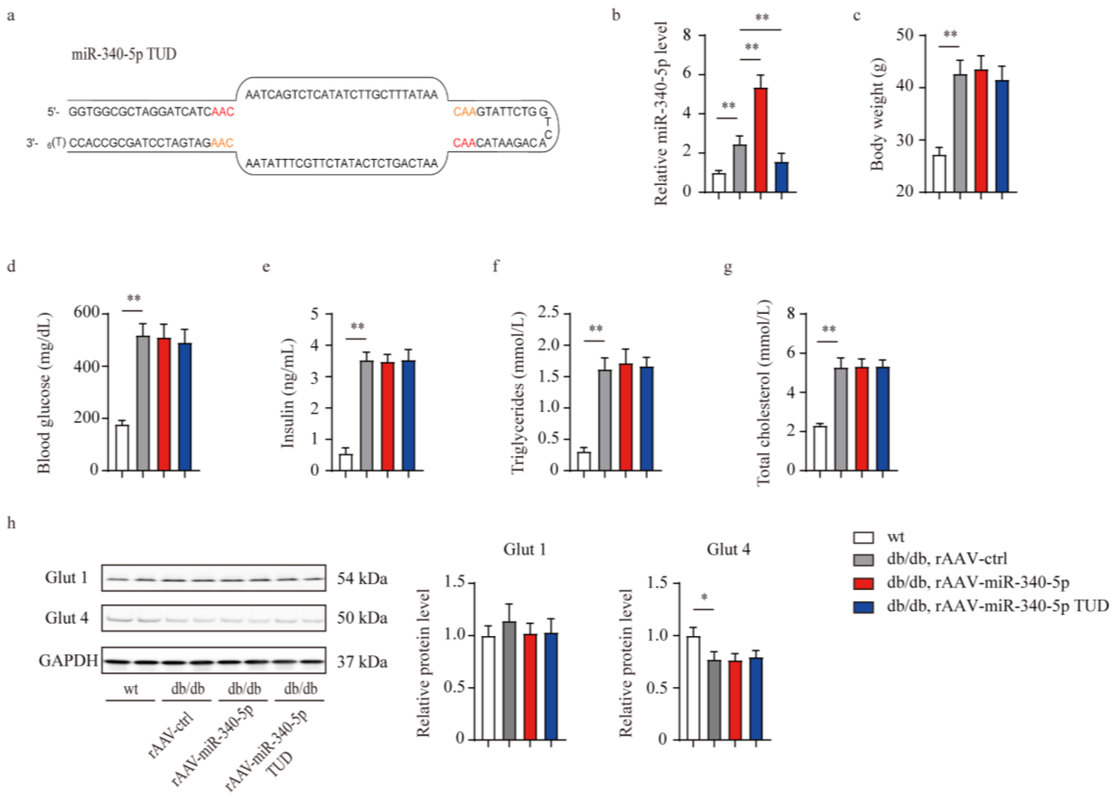

Supplementary figure 1. miR-340-5p overexpression did not affect blood glucose or lipid profile in diabetic mice.

a. Sequence of rAAV-miR-340-5p inhibitor in TUD vector. b. qRT-PCR analysis of miR-340-5p levels in heart tissues of mice at 30-week-old (n=8 in each group). c. Body weight in wt and db/db mice transfected with different rAAV9 vectors (n=8 in each group). d-g. Fasting blood glucose (d), plasma insulin concentration (e), triglycerides (f) and total cholesterol (g) were measured in wt and db/db mice transfected with different rAAV9 vectors (n=8 in each group). h. Western blot analysis and quantification of Glut 1 and Glut 4 expression in heart tissues as well as quantification of each group of mice. n=6 in each group. Data were represented as the mean±SEM. Comparison determined with one-way ANOVA followed by Tukey-Kramer test, *P<0.05 and **P<0.01.

Supplementary figure 2

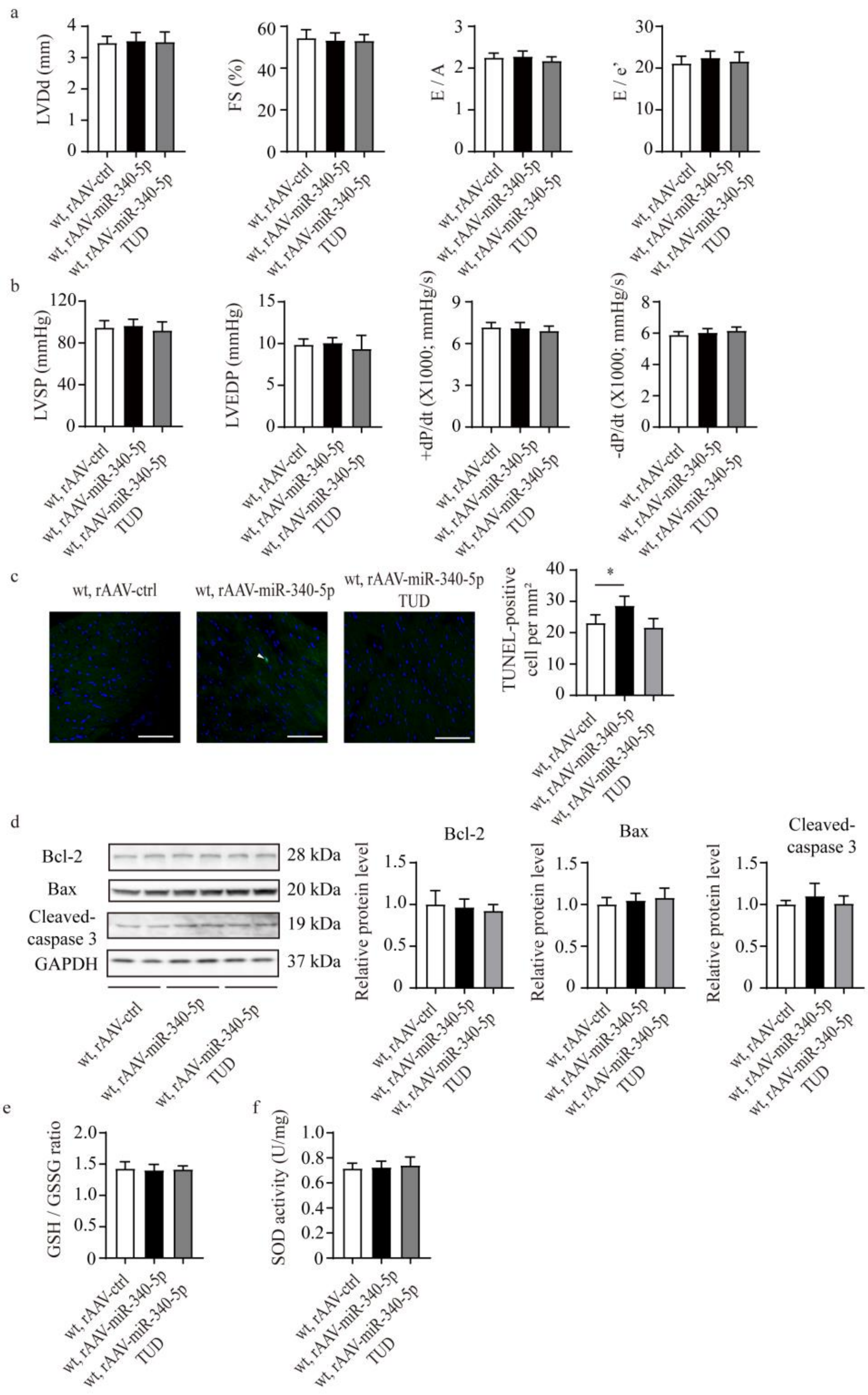

Supplementary figure 2. Measurement of cardiac function and apoptosis in heart tissues of wt mice.

a. M-mode echocardiography examination of 30-week-old wt under rAAV-miR-340-5p or rAAV-miR-340-5p TUD treatment. Left ventricular end-diastolic diameter (LVDd), FS% (fractional shortening), E/A ratio, and E/e′ ratio. n=6 in each group of mice. b. Hemodynamic

analysis of 30-week-old wt mice under different treatments. Left ventricular systolic pressure (LVSP), left ventricular end-diastolic pressure (LVEDP), +dP/dt, and –dP/dt were measured. n=6 in each group of mice. c. Representative images and quantitative analysis of TUNEL -stained heart sections of wt mice. Scale bar, 100 μm. n=5-6 in each group. d. Western blot analysis of Bcl-2, Bax and cleaved caspase 3 expressions in heart tissues as well as quantification of each group of mice. n=6 in each group. e and f. Oxidative stress in the myocardial tissues was determined by measuring the glutathione (GSH) to oxidized glutathione (GSSG) ratio and superoxide dismutase (SOD) activity of each group of mice. n=6 in each group. Data were represented as the mean±SEM. Comparison determined with one-way ANOVA followed by Tukey-Kramer test, *P<0.05.

Supplementary figure 3

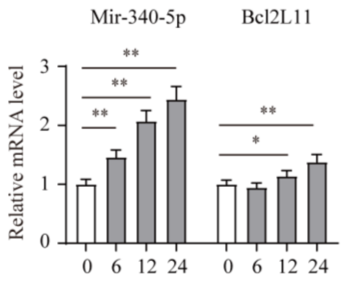

Supplementary figure 3. Increased Bcl2L11 expression in diabetic condition.

Time course analysis of expression of miR-340-5p and Bcl2L11 in HL-1 cells treated with high glucose (30 mmol/L) and palmitate (0.5 mmol/L), the expression levels were determined by qRT-PCR (n=4 independent experiments). Data were represented as the mean±SEM. Comparison determined with one-way ANOVA followed by Tukey-Kramer test. *P<0.05 and **P<0.01 vs 0 h post-treatment.

Supplementary figure 4

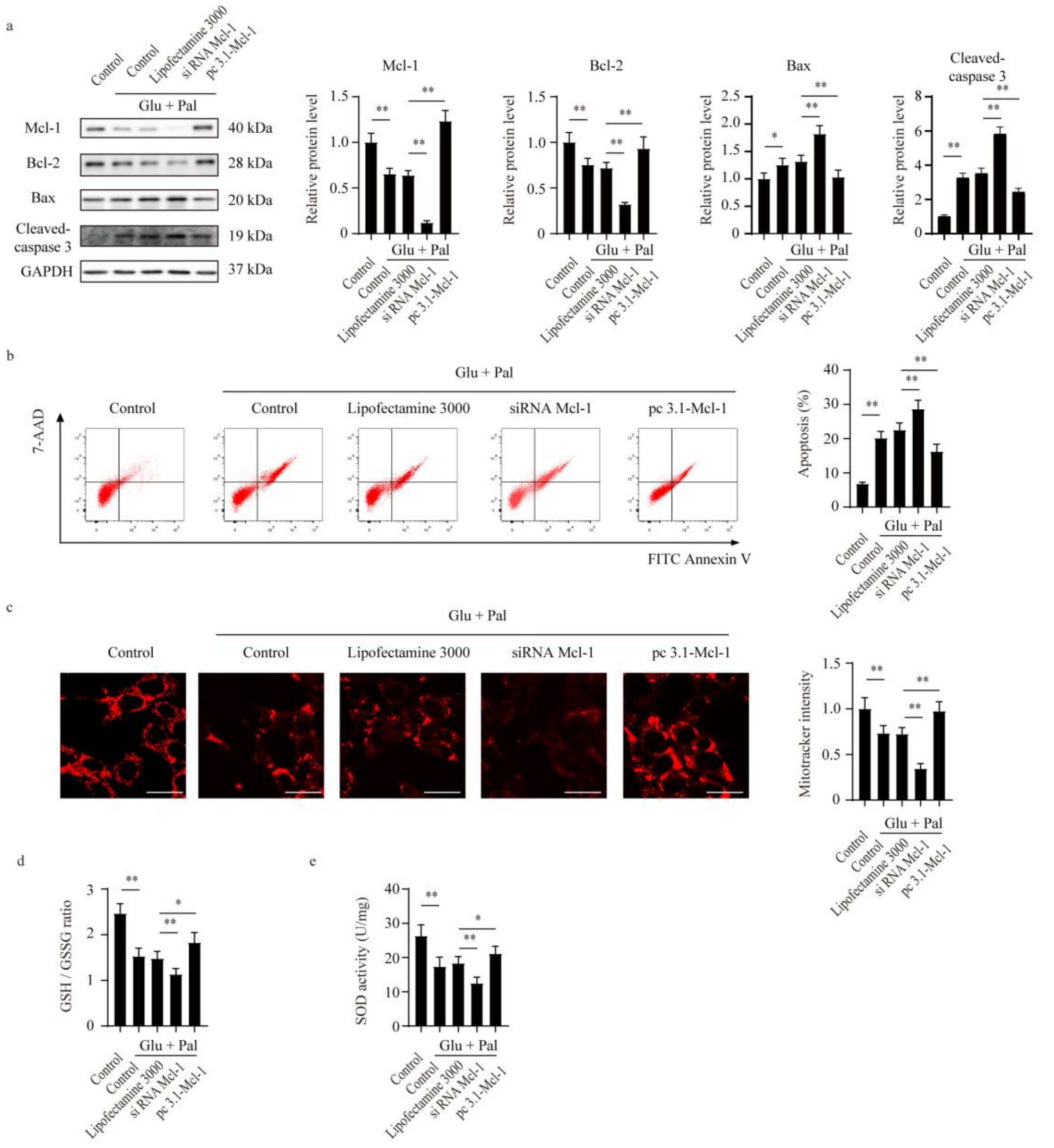

Supplementary figure 4. Mcl-1 alleviates diabetic condition -induced apoptosis and mitochondrial injury.

a. Western blot analysis of Mcl-1, Bcl-2, Bax and cleaved caspase 3 expressions in treated HL-1 cells under control or high glucose (30 mM)/palmitate (0.5 mM) conditions. b. The apoptosis was measured by flow cytometry in HL-1 cells treated under diabetic condition for 6 hours after different treatment. c-e. HL-1 cells were cultured in different treatment for 48 hours. MitoTracker staining and intensity analysis (c, Scale bar, 10 μm), GSH to GSSG ratio (d) and SOD activity (e) were determined. n=4 independent experiments. Data were represented as the mean±SEM. Comparison determined with one-way ANOVA followed by Tukey-Kramer test. *P<0.05 and **P<0.01.

Supplementary figure 5

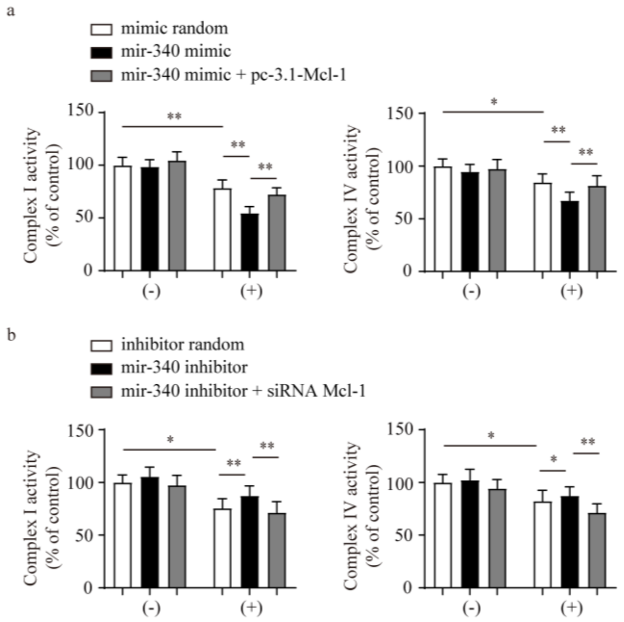

Supplementary figure 5. miR-340-5p regulates mitochondrial dysfunction by targeting Mcl-1. a. HL-1 cells were cultured under control or high glucose (30 mM)/palmitate (0.5 mM) conditions after transfection with the miR-340 mimic with or without the pcDNA 3.1(+)-Mcl-1 plasmid. Complex I activity and complex IV activity were measured and presented as percentage of control (n=4 independent experiments). b. HL-1 cells were cultured under control or high glucose (30 mM)/palmitate (0.5 mM) conditions after transfection with the miR-340 inhibitor with or without the siRNA Mcl-1. Complex I activity and complex IV activity were measured and presented as percentage of control (n=4 independent experiments). Data were represented as the mean±SEM. Comparison determined with one-way ANOVA followed by Tukey-Kramer test, *P<0.05 and **P<0.01.

Table S1. Clinic characteristics of patients in this study.

	**Non-diabetic patients （n=39）**	**Diabetic patients （n=110）**	**P value**
Male, n (%)	23 (59.0)	64 (58.1)	0.622
Age, years	61.46 ± 10.03	62.31 ± 10.42	0.890
Body mass index, kg/m^2	23.41 ± 3.54	23.83 ± 3.26	0.070
Smoking, n (%)	19 (48.7)	51 (46.3)	0.407
Hypertension, n (%)	26 (61.5)	62 (56.4)	0.012
SBP, mmHg	132.19 ± 19.53	136.46 ± 21.06	0.381
DBP, mmHg	73.68 ± 12.54	79.45 ± 15.20	0.082
Fasting glucose, mmol/L	4.87 ± 0.55	6.94 ± 0.72	< 0.001
HbA1c, %	5.71 ± 0.57	7.28 ± 0.68	< 0.001
Triglyceride, mmol/L	1.25 ± 0.39	1.31 ± 0.44	0.138
Total cholesterol, mmol/L	3.92 ± 1.02	4.05 ± 1.06	0.246
HDL cholesterol, mmol/L	1.08 ± 0.27	1.02 ± 0.25	0.047
LDL cholesterol, mmol/L	2.43 ± 0.82	2.48 ± 0.87	0.378
Uric acid, μmol/L	363.87 ± 92.63	380.30 ± 114.64	0.023
Blood urea nitrogen, mmol/L	5.50 ± 0.91	6.60 ± 1.02	<0.001
Serum creatinine, μmol/L	78.84 ± 8.51	84.84 ± 8.82	0.065
eGFR, mL/min/1.73 m^2	78.12 ± 16.15	74.34 ± 17.46	0.074
hsCRP, mg/L	1.24 ± 0.84	1.46 ± 0.91	0.067

Values are given as mean ± standard deviation or number (percentage). SBP, systolic blood pressure; DBP, diastolic blood pressure; HbA1c, glycated hemoglobin A1c; HDL, high-density lipoprotein; LDL, low-density lipoprotein; eGFR, estimated glomerular filtration rate; hsCRP, high-sentivity C-reactive protein.

Table S2. Cardiac functions measured by echocardiography in diabetic patients

	**Diabetic patients no HF （n=62）**	**Diabetic patients HF （n=48）**	**P value**
NT-proBNP, pg/mL	193.20 ± 64.15	1295.60 ± 328.60	<0.001
LVEDD, mm	49.4 ± 3.45	64.7 ± 4.35	<0.001
LVESD, mm	32.1 ± 3.05	54.5 ± 3.80	<0.001
LVEDV, mL	119.0 ± 14.85	204.0 ± 36.45	<0.001
LVESV, mL	42.5 ± 6.30	119.0 ± 15.30	<0.001
LVEF, %	55.7 ± 4.80	34.6 ± 3.25	<0.001

Values are given as mean ± standard deviation. NT-proBNP, N-terminal pro-B-type natriuretic peptide; LVEDD, left ventricular end-diastolic diameter; LVESD, left ventricular end-systolic diameter; LVEDV, left ventricular end-diastolic volume; LVESV, left ventricular end-systolic volume; LVEF, left ventricular ejection fraction.

Table S3. Changed genes in diabetic heart compared with control and filtered according to the predicted miR-340-5p target interactions.

Gene symbol	FPKM (vs control)
Slc25a3	1422.1
Lpl	1081.2
Fhl2	900.6
Uba52	567.9
Idh2	546.9
Cd36	515.4
Ech1	451.1
Acadm	394.7
Mrpl42	352.6
Hspa8	345.1
Hadh	337.6
Sdhb	296.1
Nnt	261.9
Psap	247.8
Rpl32	215.8
Pdk2	193.9
Cox17	182.9
ETFDH	176.4
Pdlim5	152.7
Hspa9	132.4
Msi2	111.3
Itm2b	99.6
Chpt1	84.9
Pgm2	82.8
Itgb1	76.6
Rilpl1	72.6
Camta1	64.5
Ppp1r12b	64.1
Ddx5	59.7
Ndrg4	56.7
Phtf2	54.6
Lrrc14b	49.3
Pgk1	47.6
Mcl1	44.7
Eif4e	43.6
Pdk1	40.6
Gstm2	38.0
Vcl	37.5
Pgap2	36.2
Tsc2	35.5
SNORA12	34.8
Acot2	34.6

Pcdh7	33.5
Snd1	32.7
Npm1	31.4
Ubl3	31.4
Kcnh2	30.0
Rplp2	29.2
Bcl2l11	29.1
Prnp	28.4
Btbd1	27.9
Tufm	26.7
Trim35	26.5
Tmem135	26.2
Laptm4b	26.1
Psmb5	26.0
Gnb2	25.8
Kars	25.8
Asb15	25.7
Son	25.1
Gpx1	24.7
1810013D10Rik	24.5
Glg1	24.1
Ktn1	23.7
Ppp3r1	23.6
Fkbp8	23.5
Mgst1	23.5
Gns	23.2
Cux1	22.8
Mrpl33	22.7
1500003O03Rik	22.4
Cdc37l1	22.0
Mrps18a	21.8
Snord15a	21.5
Efnb3	21.4
Ensa	20.8
Synpo	20.7
Rdx	20.4
Rab28	20.4
Galnt1	20.3
Tmed7	20.0
Mccc2	20.0

Full scans of western blotting data shown in Figures

Uncropped menbranes for Fig. 3b

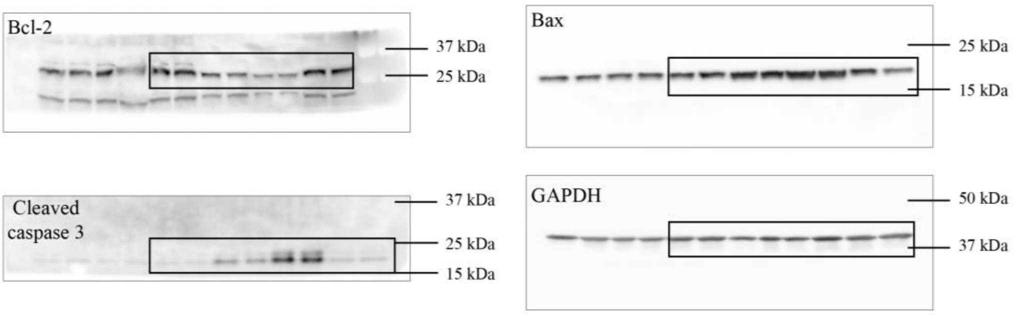

Uncropped menbranes for Fig. 4f

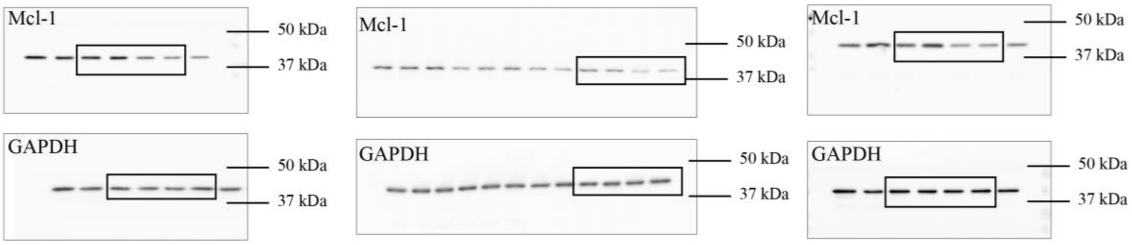

Uncropped menbranes for Fig. 4j

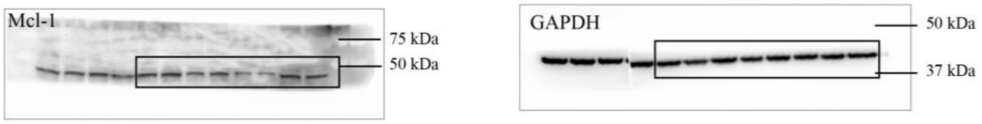

Uncropped menbranes for Fig. 5a

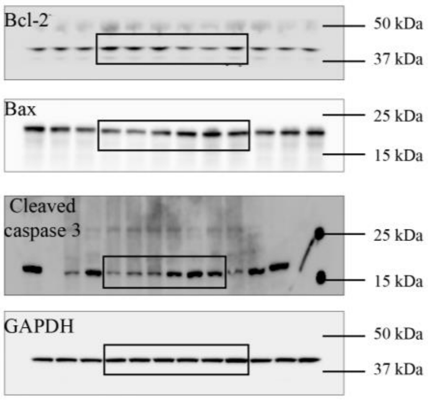

Fig. 5c

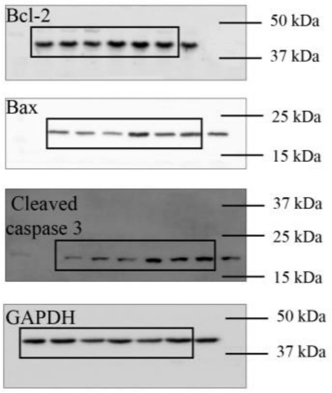

Full scans of western blotting data shown in Supplementary figures

Uncropped membranes for
Supplementary Figure 1a

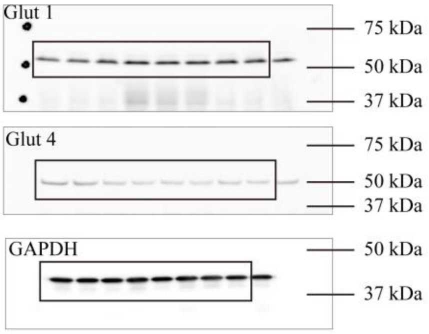

Uncropped membranes for
Supplementary Figure 2d

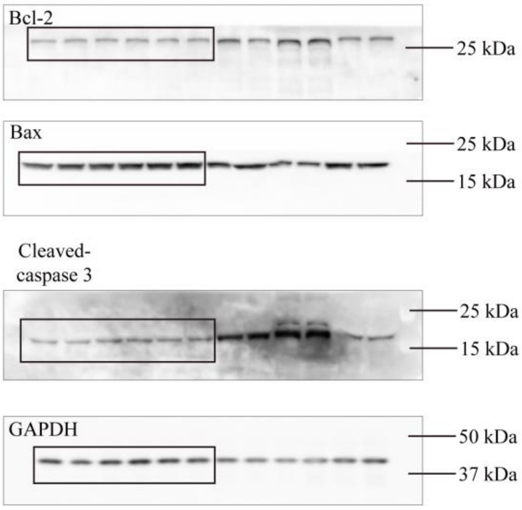

Uncropped membranes for
Supplementary Figure 4a

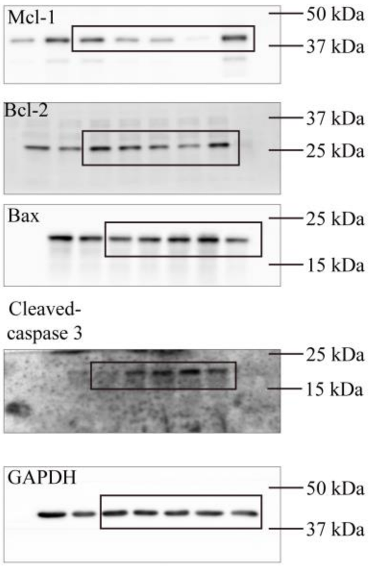

Supplement: Supplementary Materials — Supplementary Figure 1: miR-340-5p overexpression did not affect blood glucose or lipid profile in diabetic mice. (a) Sequence of rAAV-miR-340-5p inhibitor in TUD vector. (b) qRT-PCR analysis of miR-340-5p levels in heart tissues of mice at 30 weeks old (n = 8 in each group). (c) Body weight in wt and db/db mice transfected with different rAAV9 vectors (n = 8 in each group). (d–g) Fasting blood glucose (d), plasma insulin concentration (e), triglycerides (f), and total cholesterol (g) were measured in wt and db/db mice transfected with different rAAV9 vectors (n = 8 in each group). (h) Western blot analysis and quantification of Glut 1 and Glut 4 expression in heart tissues as well as quantification of each group of mice. n = 6 in each group. Data were represented as the mean ± SEM. Comparison determined with one-way ANOVA followed by the Tukey-Kramer test. ∗P < 0.01. Supplementary Figure 2: measurement of cardiac function and apoptosis in heart tissues of wt mice. (a) M-mode echocardiography examination of 30-week-old wt under rAAV-miR-340-5p or rAAV-miR-340-5p TUD treatment. Left ventricular end-diastolic diameter (LVDd), FS% (fractional shortening), E/A ratio, and E/e′ ratio. n = 6 in each group of mice. (b) Hemodynamic analysis of 30-week-old wt mice under different treatments. Left ventricular systolic pressure (LVSP), left ventricular end-diastolic pressure (LVEDP), +dP/dt, and −dP/dt were measured. n = 6 in each group of mice. (c) Representative images and quantitative analysis of TUNEL-stained heart sections of wt mice. Scale bar: 100 μm. n = 5 − 6 in each group. (d) Western blot analysis of Bcl-2, Bax, and cleaved caspase 3 expressions in heart tissues as well as quantification of each group of mice. n = 6 in each group. (e, f) Oxidative stress in the myocardial tissues was determined by measuring the glutathione (GSH) to oxidized glutathione (GSSG) ratio and superoxide dismutase (SOD) activity of each group of mice. n = 6 in each group. Data were represent [file 3182931.f1.pdf]
